# Fecal diversion does not support healing of anus-near pressure ulcers in patients with spinal cord injury—results of a retrospective cohort study

**DOI:** 10.1038/s41393-021-00717-2

**Published:** 2021-10-07

**Authors:** Andreas M. Pussin, Luisa C. Lichtenthäler, Mirko Aach, Thomas A. Schildhauer, Thorsten Brechmann

**Affiliations:** 1 Gastroenterology and Hepatology, Berufsgenossenschaftliche Universitätsklinik Bergmannsheil Bochum gGmbH, Bochum, Germany; 2 Department of General and Trauma Surgery, Spinal Cord Injury Unit, Berufsgenossenschaftliche Universitätsklinik Bergmannsheil Bochum gGmbH, Bochum, Germany; 3 Department of General and Trauma Surgery, Berufsgenossenschaftliche Universitätsklinik Bergmannsheil Bochum gGmbH, Bochum, Germany

**Keywords:** Trauma, Outcomes research

## Abstract

**Study Design:**

Retrospective cohort study including spinal cord injured patients with anus-near pressure ulcers.

**Objective:**

The primary objective was to evaluate the impact of stool diversion via stoma on the decubital wound healing. Secondary objectives included the risk of complications and ulcer recurrence. Associations between the wound healing and potentially interfering parameters were determined.

**Setting:**

University hospital with a spinal cord injury unit.

**Methods:**

A total of 463 consecutive patients who presented with a decubitus were retrospectively included. Patients with and without a stoma were compared using descriptive and explorative statistics including multiple regression analysis.

**Results:**

The severity of the pressure ulcers was determined as stage 3 in two-thirds and stage 4 in one-third of all cases. The wound healing lasted longer in the 71 stoma-presenting patients than in the 392 patients with undeviated defecation (77 vs. 59 days, *p* = 0.02). The age (regression coefficient *b* = 0.41, *p* = 0.02), the ASA classification (*b* = 16.04, *p* = 0.001) and the stage of the ulcers (*b* = 19.65, *p* = 0.001) were associated with prolonged ulcer treatment in the univariate analysis. The multiple regression analysis revealed that the fecal diversion (*b* = −18.19, *p* = 0.03) and the stage of the ulcers (*b* = 21.62, *p* = 0.001) were the only predictors of delayed wound healing.

**Conclusion:**

The presence of a stoma is not related to improved wound healing of ulcers near the anus. On the contrary, stoma patients needed more time until complete wound healing, conceivably related to selection bias. Nonetheless, we currently do not recommend fecal diversion to be the standard concept for decubitus treatment.

## Introduction

Pressure skin ulcers are a common complication and reason for hospitalization in patients with spinal cord injury (SCI) leading to a huge medical, social, and economic burden [1, 2]. The healthcare costs for the treatment of pressure ulcers in SCI patients are significantly higher than the costs for those without pressure ulcers, partly explained by longer inpatient care [3]. Whereas first-degree pressure ulcers predominantly in the sacral region dominate within the first 30 days after the initial trauma [4], the risk remains high and varies between 40 and 60 % in the long-term [5, 6], covering all different stages of severity; about one-third present with stage 1, about 40 % with stage 2, and a further 28 % with stage 3 or 4 [5].

Timely care and treatment can reduce the exacerbation of pressure ulcers [7]. The best current preventive approach consists of a risk-adapted lifestyle, including pressure relief, avoidance of promoting risk factors, such as nicotine abuse or alcohol intake, and careful skin monitoring [8]. The concomitant neurogenic bowel dysfunction, fecal incontinence, and defecation difficulties of patients with SCI are assumed to further aggravate the risk, particularly of sacral ulcerations [6, 9, 10]. Whereas data regarding those with SCI are missing, fecal incontinence increases the probability of ulcers in the general adult hospitalized population by twenty-two times [11].

Once the ulcer has established, the feces are assumed to deteriorate the healing process [12]. Limited data deriving from a cohort of bedbound patients support better healing and decreased recurrences after colostomy [13]. Therefore, some centers tend to deviate the feces by means of a stoma in order to reduce the risk of contamination. On the other hand, the bowel habit improves over the first six months after injury [14]. Ongoing conservative measures help to control and improve bowel movements [15], whereas the stoma construction cannot completely eliminate secretion and unconscious defecation of residual feces. Additionally, both the surgical procedure and the stoma itself may induce adverse events, such as infections, wound healing disorders, peristomal skin disorders, hernias, or psychological barriers [16]. Up to now, no study has addressed the rationale and risk-to-benefit assessment of this fecal diversion concept in SCI patients. Hence, as primary objective we aimed to investigate the association between the presence of a stoma and the healing process of stage 3 and 4 pressure ulcers in a retrospective cohort study. Secondary objectives were to evaluate the need for revision surgery, the risk of complications, and the duration of intensive care unit stay, and investigated associations between the wound healing and the etiology of the SCI, the localization, the severity, and the age of patients.

## Patients and methods

### Study population and definitions

We conducted a retrospective cohort study of consecutive SCI patients who had been hospitalized for surgical pressure ulcer treatment in the SCI Unit of the University Hospital Bergmannsheil Bochum, Germany, between 2007 and 2017. All adult patients with chronic SCI (for at least 6 months) and a surgical treatment of decubitus stage 3 or 4 according to the National Pressure Ulcer Advisory Panel (NPUAP), European Pressure Ulcer Advisory Panel (EPUAP), and Pan Pacific Pressure Injury Alliance (PPPIA) classification [17] close to the anus were regarded suitable for inclusion.

Regarding patients with multiple treatments, only the first case admitted to our hospital was considered. The severity of the SCI was classified according to the International Standards for Neurological Classification of Spinal Cord Injury (ISNCSCI) of the American Society of Spinal Cord Injury (ASIA) including the ASIA Impairment Scale (AIS) [18]. Only patients with AIS stage A or B were included.

### Clinical data acquisition

Every case was thoroughly reconstructed based on the electronic data system. Data collection included SCI characteristics such as etiology, localization, and severity, basic demographic characteristics, stoma characteristics, ulcer characteristics (such as size and stage), and surgical outcome parameters (such as healing time, number of surgical revisions, and complications).

### Definition of ulcer characteristics and staging of disability

The anus-near localization was defined as any ulcer that spread out with at least a substantial surface within the following anatomical structures: The connecting line between both posterior superior iliacal spines formed the cranial margin, the ventrocaudal margin was compartmentalized by the connecting lines between the two ischial tuberosities and the penis root, and the connecting line between the posterior superior iliacal spines and the ischial tuberosities demarcated the lateral margins. The severity level of the ulcers was classified according to the EPUAP, NPUAP, and PPPIA [17]. The healing was evaluated based on the clinical charts; according to standard care patients were discharged with a completely healed ulcer. Therefore, if the detailed description of the achievement of the complete wound healing time was not available, the time of discharge was defined as the endpoint of wound healing. Complications that occurred during ulcer treatment were divided into five severity degrees (Table 1S). A primary colonization was defined by the proof of any microbial species within the pressure ulcer at admission; a subsequent infection with a previously undiagnosed germ was considered as a superinfection. The overall morbidity and periprocedural risk were categorized by the American Society of Anesthesiologists (ASA) score that distinguishes between healthy persons, subjects with mild systemic disease, subjects with severe systemic disease, subjects with severe systemic disease that is a constant threat to life, and moribund persons who are not expected to survive without the operation.

### Standard surgical procedure

Surgical procedures, including the mechanical wound debridement with removal of coatings and necrotic tissue, were realized under general anesthesia. Fascio-cutaneous flaps were typically used to cover tissue defects in areas close to the anus. The fascia was mobilized after skin incision and cutting of the subcutaneous fat by thermocautery. A bridge of skin was kept and the flap was transposed without tension. Redon drains were placed and the flap was fixed in the center with a single button suture. The lateral margins were closed with subcutaneously adapted single button sutures under low tension. Skin sutures were realized by Donati’s back-and-forth technique. Finally, a wound dressing and sterile bandage were placed. The therapeutic standard did not change during the observation period; vacuum therapy has never been established as a standard therapy.

### Statistics

Statistical analysis was performed with SPSS Version 26 (IBM, Armonk, USA). Categorial parameters were stated as absolute values and relative frequencies. Median and interquartile range (IQR) were used for the evaluation of metric variables. If necessary, quantitative variables were transferred into numerical categories. Explorative statistics included ANOVA, Fisher’s exact test, chi-squared test with Yates correction, two-tailed *t* test, Mann–Whitney *U* test, and univariate and multivariate regression analysis. In this model, the dummy variables created for the ASA classification led to the exclusion of ASA grade 4 due to a lack of correlation. Results were considered statistically significant with a *p* value ≤ 0.05.

### Ethical considerations

The study protocol has been reviewed and approved by the institutional review board of the Ruhr-University Bochum [registry number 18-6351] based on the ethical guidelines of the Declaration of Helsinki and its later revisions. Written, informed consent was obtained from all patients before surgery. Informed consent was neither practicable nor necessary due to its retrospective character.

## Results

### Study population

A total of 463 SCI patients (111 women (24.0 %), median age 53 years (IQR 43—64 years)) of whom 71 patients (15.3 %) carried a stoma were included (Table 2S). The median duration of the SCI was 222 (76–382) months. Traumatic events were responsible for more than half of the disabilities (60.7 %). Most patients (*n* = 402, 86.8 %) presented AIS stage A. There were slight differences between the study groups regarding the etiology and severity of the SCI with higher grades in the fecal diversion group.

**Fig. 1:**
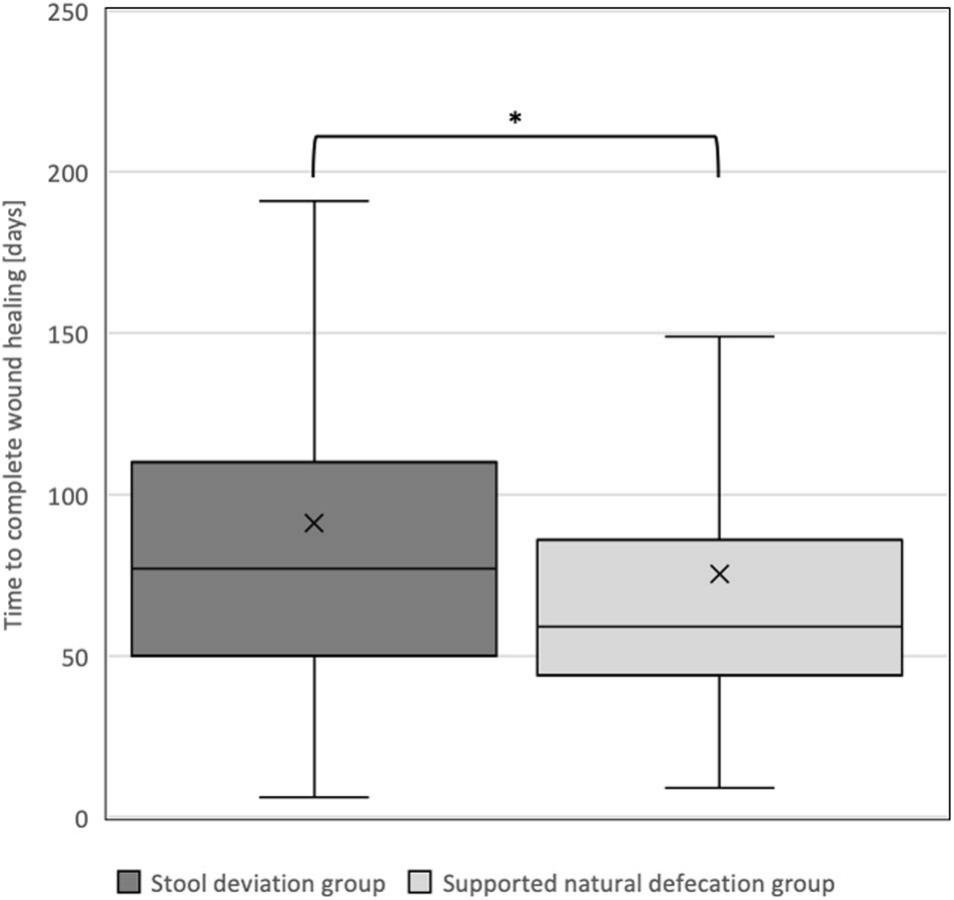
Duration of complete wound healing. This box plot diagram illustrates that the completion of wound healing lasted significantly longer in patients with fecal diversion than in the natural defecation group (— median value 77 vs. 59 days [*x*: arithmetic mean 92 vs. 72 days], * *p* < .05). Similarly, the interquartile range varied more widely in the fecal diversion group ([50; 110] vs. [44; 86]).

The body mass index was slightly but significantly higher in the stoma presenting patients (26.3 (22.1–29.4) kg/m^2^ vs. 24.2 (20.5–27.4) kg/m^2^, *p* = 0.04). The presence of at least one comorbidity differed similarly (95.8 vs. 86.2 %, *p* = 0.02) (Table 1) with a comparable distribution (Table 3S and Fig. 1S). Almost all patients presented as ASA stage 2 (45.8 %) or 3 (52.9 %). The staging differed significantly between the study groups (64.8% ASA stage 3 in the fecal diversion group vs. 50.8 %) (Table 1 and Fig. 2S). The medication did not distinguish (Table 4-S). The red blood cell count (Table 5S) was significantly lower among the stoma-treated patients both prior to surgery (10.9 vs. 12.1 g/dl, *p* = 0.00005) and at discharge (11.2 vs. 12.2 g/dl, *p* = 0.007).

**Table 1:** Basic demographic characteristics.

		Number (%), Average or Median (IQR)^†^		
	*n*	Fecal diversion group	Supported natural defecation group	*p* value^††^
**Gender** [female]	71/392	14 (19.9)	97 (24.7)	0.36
**Age** [years]	71/392	54 (44; 65)	53 (43; 63)	0.54
**Body mass index** [kg/m^2^]	71/381	26.3 (22.1; 29.4)*	24.2 (20.5; 27.4)	0.04
**Duration since SCI** [months]	59/346	280 (97; 406)	219 (71.8; 380.3)	0.11
Aetiology of SCI**	69/362			–
Trauma		36 (52.2)	245 (67.7)	
Infection		2 (2.9)	10 (2.8)	
Neoplasia		0 (0)	13 (3.6)	
Other		31 (44.9)	94 (26.0)	
**ASIA classification**	68/383			0.56
Stage A		62 (91.2)	340 (88.8)	
Stage B		6 (8.8)	43 (11.2)	
Stage C		0 (0)	0 (0)	
Stage D		0 (0)	0 (0)	
Stage E		0 (0)	0 (0)	
**Comorbidities**	71/391	68 (95.8)*	337 (86.2)	0.02
**Smoking habit**	60/346			0.64
Active smoker		17 (28.3)	88 (25.4)	
Non- smoker		43 (71.7)	258 (74.6)	
**Alcohol consumption**	71/392	5 (8.5)	25 (7.4)	0.76
**ASA classification**^††^**	71/392			0.002
Stage 1		0 (0)	0 (0)	
Stage 2		22 (31)	190 (48.5)	0.004
Stage 3		46 (64.8)	199 (50.8)	0.03
Stage 4		3 (4.2)	3 (0.8)	0.002
Stage 5		0 (0)	0 (0)	
**Presence of a stoma**	71/392	71 (100)	0 (100)	–
End-Colostomy		26 (36.6)	0 (0)	
Loop-Ileostomy		17 (23.9)	0 (0)	
**Time between stoma surgery and current ulcer** [months]	29	40 (2.3; 156.1)	n/a	–
**Adverse event during stoma surgery**	44	11 (25)	n/a	–
**Previously surgical until wound healing**	66/379	1 (0; 2)	1 (0; 2)	0.31
**Previously surgical ulcer procedures**	71/392	39 (54.9)	207 (52.8)	0.74
**Severity Ulcer**	71/392	71 (100)*	392 (100)	0.02
Stage 3		36 (50.7)	255 (65.1)	
Stage 4		35 (49.3)	137 (34.9)	
**Size Ulcer** [cm^2^]	60/329	25 (10.5; 71.5)*	20 (9; 35)	0.04

*ASA* American Society of Anesthesia, *ASIA* American Spinal Injury Association, *SCI* spinal cord injury.

^†^as appropriate.

^††^regardless SCI. Chi2-Test or Mann–Whitney *U* test, as appropriate.

*significant with *p* < 0.05. **significant with *p* < 0.01.

### Baseline stoma characteristics

Twenty-six (36.6 %) of the 71 patients with a stoma had been treated with an end colostomy and 17 patients (23.9 %) with a loop ileostomy. The stoma characteristics for the remaining 28 patients were not documented accurately. There was an average of 40 months (IQR 2.3–156.1) between stoma surgery and admission for ulcer treatment. Eleven of the stoma-treated patients (25.0 %) had suffered relevant complications during stoma surgery (Table 6-S). The manual evacuation was necessary in 156 patients without a stoma and in only one with a stoma. Sixty-two stoma patients needed neither manual evacuation nor topical or systemic laxatives.

### Baseline ulcer characteristics

The average size of the ulcerations was an estimated 16.0 (8.0– 30.0) cm^2^. About 291 of the patients presented a stage 3 and 172 (37.1 %) a stage 4 decubitus. A total of 246 patients had surgically been pretreated in the same area. At least one pathogen could be detected in 434 patients (93.7 %) at the start of treatment (Table 7-S).

### Outcome of ulcer treatment

The time to complete wound healing in 445 patients had to be equated with the time of discharge and the healing could not described in 18 patients because they either died in hospital (*n* = 11) or there was no documentation of the length of the hospital stay (*n* = 7). Prior ulcer treatment did not differ between the two groups. The median duration of ulcer treatment until the wound healed completely amounted to 61 days (IQR 44–89, *n* = 445) (Table 7S). The stoma group needed an average, though not significantly, of ten days more after the first ulcer perception to surgery than the non-deviating group (70 vs. 60 days, *p* = 0.64) (Table 2). The length of hospital stay was significantly longer among the stoma-treated patients (78 vs. 59 days, *p* = 0.003), whereas the need for intensive care treatment was quite similar (2.5 days in the stoma group vs. 4.5 days, *p* = 0.65). Stoma patients needed 77 days (IQR 50–110) to complete wound healing, whereas those without a stoma needed 59 days (IQR 44–86) (*p* = 0.02) (Fig. 1). In a subgroup analysis with a limited number of cases the nature of the stoma did not influence the outcome (Table 3).

**Table 2:** Outcome parameters.

		Number (%) or Median (IQR)^†^		
	*n*	Fecal diversion group	Supported natural defecation group	*p* value^††^
**Admission to surgery time** [days]	39/201	70 (28; 130)	60 (27; 131)	0.64
**Primary microbial infection**	71/392	65 (91.5)	369 (94.1)	0.41
**Microbial superinfection**	69/382	12 (17.4)	83 (21.7)	0.42
**Time to complete wound healing** [days]	66/379	77 (50; 110)*	59 (44; 86)	0.02
**Length of hospital stay** [days]	71/390	78 (50; 118)**	59 (43; 86)	0.003
**Length of ICU stay** [days]	71/392	2.5 (2; 34.5)	4.5 (3.25; 22)	0.65
**Adverse events during ulcer treatment**	71/392	39 (54.9)	206 (52.6)	0.71
Stage 1		1 (2.6)	14 (6.8)	
Stage 2		26 (66.7)	150 (72.8)	
Stage 3		3 (7.7)	13 (6.3)	
Stage 4		6 (15.4)	21 (10.2)	
Stage 5		3 (7.7)	8 (3.9)	
**Adverse events during stoma surgery**	44	11 (25)	n/a	
**Adverse events in total**	71/392	48 (67.6)	221 (56.4)	0.08
**Clostridioides difficile infection**	71/392	2 (2.8)	8 (2.0)	0.68
**Recurrence** [within 3 month]	71/392	2 (2.8)	11 (2.8)	1.00
**New ulcer in the same region** [within 3 months]	71/392	7 (9.9)	23 (5.9)	0.21
**Recurrence in total**	71/392	9 (12.7)	34 (8.7)	0.40

^†^as appropriate.

^††^regardless SCI. Chi^2^-test or Mann–Whitney *U* test, as appropriate.

*significant with *p* < 0.05.

**significant with *p* < 0.01

**Table 3:** Subgroup analysis regarding stoma types.

		Number (%) or Median (IQR)^†^		
	*n*	End colostomy *n* = 26	Loop ileostomy *n* = 17	*p* value^††^
**Admission to surgery time** [days]	17/7	50 (23; 90)	90 (42; 90)	0.52
**Previously surgical ulcer procedures**	26/17	14 (53.8)	11 (64.7)	0.54
**Primary microbial infection**	26/17	26 (100)	15 (88.2)	0.15
**Microbial superinfection**	25/17	5 (20)	2 (11.8)	0.78
**Time to complete wound healing** [days]	22/17	72.5 (50; 110)	92 (49; 179)	0.36
**Length of hospital stay** [days]	26/17	75 (50; 116)	98 (49; 179)	0.42
**Length of ICU stay** [days]	26/17	2 (2; 13)	4 (2; 2)	0.52
**Adverse events during ulcer treatment**	26/17	15 (57.7)	8 (47.1)	0.71
**Adverse events during stoma surgery**	23/14	3 (13)	4 (28.6)	0.39
**Adverse events in total**	26/17	19 (73.1)	11 (64.7)	0.81
**Clostridioides difficile infection**	26/17	1 (3.9)	0 (0)	1.00
**Recurrence** [within 3 months]	26/17	1 (3.9)	1 (5.9)	1.00
**New ulcer in the same region** [within 3 months]	26/17	3 (11.5)	2 (11.8)	1.00

^†^as appropriate.

^††^Easy Fisher Exact Test, Chi^2^-test with Yates correction or Mann–Whitney *U* test, as appropriate.

Ulcer treatment-related complications occurred similarly frequently in both groups. The overall complication rate that included stoma-related adverse events differed tendentially but not significantly (48 complications (67.6 %) in patients with stoma vs. 221 (56.4 %), *p* = 0.08). Thirty-nine subjects (8.4 %) developed an ulcer recurrence within three months. There were no differences between the two groups (12.7 % in the stoma group vs. 8.7 %). A total of 95 patients (20.5 %) developed a superinfection with no differences between the two groups (Table 2).

### Ulcer healing influencing factors—time to complete wound healing

In a linear regression, the age (*p* = 0.02), the ASA classification (*p* = 0.001), and the stage of the ulcers (*p* = 0.001) showed an influence on the time to complete wound healing. The previous stoma treatment (*p* = 0.03) was revealed to be significantly but inversely associated to complete wound healing (Table 4); stoma patients required a longer time to complete wound healing than the supported natural defecation group.

**Table 4:** Ulcer healing influencing factors, simple- and multiple linear regression—Time to complete wound healing [days].

	Simple linear regression		Multiple linear regression ^A^	
Parameter	Regression coefficient	*p* value (95% CI)	Regression coefficient	*p* value (95% CI)
ASA classification	16.04	0.001 [6.406; 25.668]**	9.28	0.15 [−3.259; 21.818]
Stage ulcers	19.65	0.001 [9.222; 30.068]**	21.62	0.001 [8.446; 34.793]**
Stoma	−15.79	0.03 [−30.094; −1.481]*	−18.19	0.03 [−34.792; −1.594]*
Smoker	−4.80	0.45 [−17.255; 7.654]	6.03	0.40 [−8.064; 20.114]
Age [years]	0.41	0.02 [0.066; 0.749]*	0.04	0.88 [−0.423; 0.494]
Body mass index [kg/m^2^]	0.42	0.34 [−0.444; 1.285]	0.41	0.43 [−0.625; 1.449]
Duration since SCI [months]	0.01	0.35 [−0.016; 0.044]	−0.001	0.97 [−0.033; 0.032]
Time of surgery after the onset of the ulcer	−0.001	0.86 [−0.015; 0.012]	0.03	0.07 [−0.003; 0.057]

*significant with *p* < 0.05.

**significant with *p* < 0.01.

The multiple linear regression model has no autocorrelation (Durbin–Watson statistic 1.725). The *R*² for the overall model was .141 (adjusted *R*² = 0.099), indicative of a middle goodness-of-fit, according to Cohen. The stoma and the stage of the ulcers were able to predict hourly wage (F(8, 164) = 3.354, *p* = 0.001). Therefore, stoma (*p* = 0.03) and the stage of the ulcers (*p* = 0.001) were associated with the wound healing, whereas the ASA classification (*p* = 0.15) and age (*p* = 0.88) lost their significance (Table 4).

## Discussion

This retrospective cohort study aimed to evaluate the concept of fecal diversion to support the wound healing of anus-near pressure ulcers in patients with SCI. Surprisingly, the existence of a stoma was associated with substantially prolonged treatment of the pressure ulcer. The duration until complete wound closure differed remarkably (77 vs. 59 days, respectively). This result contrasts with the findings of a retrospective study on bedridden patients in which the stoma was associated with an accelerated wound healing (3 vs. 7 months) and fewer surgical procedures [13]. De la Fuente et al. not only included a much smaller but also a completely different cohort. The SCI patients in this representative cohort were usually younger, predominantly male, exhibited divergent comorbidities, suffered from immobility but not confinement in bed, loss of sensibility, and defecation problems [19]. Additionally, denervation influences the wound healing negatively [20, 21].

The retrospective design bears certain limitations. First of all, the stoma was rarely constructed for the purpose of the current ulcer treatment and contained different types, such as loop ileostomy and end colostomy. The nature of the stoma did not influence the outcome at all in a subgroup analysis with a limited number of cases. Interestingly, the patients with a stoma were more likely to present a stage 4 ulcer, which might reflect slightly divergent patient characteristics and starting conditions. Self-evidently, the stage and size of the pressure ulcer influenced the duration of wound healing. Second, the groups were unequal in size and differed regarding particular basic characteristics. Stoma patients at inclusion presented higher ASA stages (Table 1), reflecting worse clinical performance and slightly more comorbidities. In univariate analysis, both parameters were associated with prolonged ulcer treatment; these associations have already been described by various studies in different contexts both for higher ASA stages [22–24] and comorbidities including anemia [25, 26]. The stoma patients presented slightly but significantly higher body mass index (BMI) levels (26.3 vs. 24.2 kg/m^2^); higher BMI levels were related to higher ulcer stages. Although described in previous studies [27], the BMI in this cohort of SCI patients was not associated with delayed wound healing. Third, the retrospective design results in a loss of information. The ASA classification, for instance, was only available at the time of inclusion but not of stoma construction. Additionally, the indications for stoma construction remained unclear; it might reasonably be possible that the severity of comorbidities, a lower overall physical performance, or other clinical aspects could have driven the decision in favor of the stoma. Fourth, the cohort has been collected over a whole decade in which the management might have changed slightly, although we did not find any time-specific effects.

The substantial size of the cohort, particularly regarding the rareness of SCI, allowed us to perform a dedicated multiple regression analysis that included all eventually interacting parameters. The calculations revealed that two aspects most of all were reversely associated with the success and duration of the wound healing: The stage of the ulcer (*p* = 0.001) and the presence of a stoma (*p* = 0.03). All other parameters, such as age, clinical performance reflected by the ASA score and ASIA classification, and BMI lost their significance (Table 4). The reason for this remarkable main result that finds support from similar outcomes in patients with an open pelvic fracture [28], remains speculative. Basically, the construction of a stoma is intended to prevent the wound from fecal microbiota, but it is unclear whether the resulting colonization or superinfection plays an important role in the healing process. Additionally, the fecal diversion may alter the colonic microbiota, which sometimes leads to diversion colitis [29, 30], and SCI patients suffer from anorectal alterations such as prolapse and uncontrolled secretion and defecation [9, 10], which might allow ongoing contamination and superinfection. Therefore, it seems reasonable to investigate the microbial pattern in the wound before and during treatment in future studies.

The stoma group tended to develop more complications, although not significantly; this observation was triggered exclusively by the adverse events on the occasion of the stoma construction, while complication rates during ulcer treatment did not differ. Regarding the other secondary objectives, such as the need for revision surgery, the duration of intensive care unit stay, the occurrence of sepsis and death, and ulcer relapses, were equally distributed in both groups. Additionally, neither the duration nor the pattern of the SCI was associated with the outcome of ulcer treatment. Interestingly, we could not even reproduce risk factors that have been associated with deteriorated wound healing, such as smoking [31] and alcohol consumption, in this large SCI cohort [32].

Although the construction of a stoma showed advantages, such as improved quality of life and social independence, in some studies [33, 34], we cannot recommend this concept as a standard procedure yet. Given the high frequency of anus-near pressure ulcers in SCI patients [6, 35], the potential morbidity of stoma construction [16, 36] and the rather prolonged wound healing, the additional stoma constructing surgery should rely on individual decision-making. Additionally, a prospectively designed, controlled study that randomly investigates the influence of a stoma construction, probably standardized as end colostomy, is mandatory in order to evaluate the fecal diverting strategy as a general concept to support wound healing, particularly in SCI patients.

## Conclusion

To the best of our knowledge, this is the largest study so far and the first study in patients with SCI that investigates the impact of a stoma in the context of the treatment of anus-near pressure ulcers. The results propose that the presence of a stoma does not support the healing process. Despite all limitations of a retrospective cohort study, the multiple regression analysis indicates that stoma patients need more time until complete wound healing without any other benefits regarding adverse events. Divergent underlying patient characteristics may interfere and play a substantial role. Nevertheless, the data does not support the fecal diversion improving the healing process of anus-near pressure ulcers. Regarding the potential morbidity and psychological restrictions related to the stoma construction, we currently do not recommend it to be the standard strategy and it should rather be based on individual decision-making. A randomized controlled study is mandatory to clarify the impact of the fecal diverting concept in the context of the treatment of anus-near pressure ulcers.

## Supplementary information

Supplementary Material

## Data Availability

All data generated or analyzed during this study are included in the submission and appear either in the results or in the supplementary material section. The primary data rests with the corresponding author; all authors have unlimited access. The datasets used and/or analyzed are available from the corresponding author on reasonable request.
