# Supplementary material for: Fecal diversion does not support healing of anus-near pressure ulcers in patients with spinal cord injury—results of a retrospective cohort study
Source: Spinal Cord. 2021 Oct 7;60(6):477–83. doi: 10.1038/s41393-021-00717-2 (PMC9209324; doi:10.1038/s41393-021-00717-2)
Supplement: Supplementary file 1 — Supplementary Material [file 41393_2021_717_MOESM1_ESM.pdf]

Table 1-S: Classification of the severity of complications.

Scale	Definition
Stage 1	· Bloody-serous secretion from the wound<br>· Mild secretion from the wound<br>· Ulcer<br>· Pronounced scarring due to flap-plastic covering of the ulcer<br>· Fibrinous evidence<br>· Urinary tract infection<br>· Anemia<br>· Hematoma<br>· Hematoma by Xarelto<br>· Deterioration of laboratory parameters<br>· Fever<br>· Constipation<br>· Transplanted split skin partially detached (treated conservatively)

Stage 2	· Wound healing disorder<br>· Greatly delayed wound healing<br>· Wound healing disorder due to massive diarrhea<br>· Tearing of the scar after mobilization<br>· Arrosion in the scar<br>· Recurrence<br>· Partial dehiscence of the scar and dislocation of the embedded redon drainage<br>· Wound dehiscence and postoperative anemia<br>· Wound dehiscence with fistula formation<br>· Hemorrhage with wound dehiscence<br>· Remaining highly secreting fistula<br>· New epithelial lesions and persistent inflammation<br>· Candida intertrigo inguinal spread to surgical wound<br>· Urinary tract infection with fever and chills<br>· Urinary tract infection with Proteus mirabilis (3MRGN)<br>· Postoperative local infection<br>· MRSA<br>· Superinfection 3MRGN<br>· Superinfection 4MRGN<br>· Superinfection 3MRGN and 4MRGN<br>· Allergic to antibiotics<br>· Fluid retention<br>· Postoperative desaturation<br>· Abdominal discomfort and bacterial conjunctivitis<br>· Pointed hemorrhage right ischial seat and UTI with Pseudomonas aeruginosa (3MRGN)<br>· Lymphedema<br>· Ulnar nerve syndrome<br>· Dehiscence with fistula formation and feverish UTI<br>· Severe AB-associated diarrhea and wound dehiscence<br>· Wound healing disorder and MRSA<br>· Wound dehiscence and postoperative anemia and necrosis<br>· Re-education of necrosis<br>· Suture necrosis with suture insufficiency<br>· Wound healing disorder and infection<br>· Abscess formation<br>· Ischial pressure ulcer stage 2 after surgery
Stage 3	· Postoperative pneumothorax<br>· Postoperative delirium<br>· Convulsive seizures in the course<br>· Non-neurological convulsive seizures<br>· Psychosis<br>· Reopening of the wound by traction and hemoptysis<br>· Postoperative thrombosis<br>· Deep leg vein thrombosis<br>· Neuropathic pain and abdominal pains<br>· Delayed wound healing with scar carcinoma and squamous cell carcinoma<br>· Wound healing disorder with necrosis and MRSA<br>· Bleeding

Stage 4	· Respiratory insufficiency with intubation in intensive care<br>· Postoperative pneumonia<br>· Postoperative pulmonary embolism<br>· Wound healing disorder and postoperative bleeding anemia<br>· Relocation of the right main bronchus, as well as clostridia-associated diarrhea and reanimation required for pneumothorax<br>· Postoperative systemic infection<br>· Septic shock<br>· Sepsis and allergic reactions to Octenisept and Imipenem as well as dehiscence and hemothorax<br>· Cardiopulmonary decompensation<br>· Cardiac deterioration<br>· Media Part infarction<br>· Pancreatitis with cholecystitis and UTI<br>· Agranulocytosis by beta-lactams<br>· Postoperative arterial bleeding with neutropenia and dehiscence<br>· Allergy to antibiosis and ileus<br>· Necrotizing fasciitis made wound healing difficult<br>· Catecholamine decrease and endotracheal intubation<br>· Palliative
Stage 5	· Endotracheal intubation with pneumonia and death<br>· Bleeding with necrosis and death<br>· General deterioration with renal insufficiency leading to death<br>· Brain hemorrhage with fatal consequences<br>· Postoperative intracranial hemorrhage with death<br>· Resuscitation and death by acute liver failure in cirrhosis<br>· Through-syndrome and cardio-pulmonary poor and occurrence of further decubitus at agitation and ultimately death<br>· Circulatory failure and death<br>· Septic anemia with fatal consequences<br>· Death by sepsis

Table 2-S: Baseline characteristics.

	n	Number (%) or Median (IQR)$^{+}$
**Gender** [female]	463	111 (24.0)
**Age** [years]	462	53.0 (43; 64)
**Body mass index** [kg/m^2]	452	24.6 (21; 28)
**Duration since SCI** [months]	405	222 (75.5; 382)
**Aetiology of SCI**	430	
Trauma		281 (60.7)
Infection		12 (2.6)
Neoplasia		13 (2.8)
Other^{++}		124 (26.8)
**ASIA Impairment Scale**	451	
A		402 (89.1)
B		49 (10.9)
**Comorbidities**	462	405 (87.7)
**Smoking habit**	406	
Smoker		105 (22.7)
Non-smoker/former smoker		301 (65.0)
**Alcohol consumption**	463	30 (6.5)
**ASA classification**$^{+++}$	463	
Stage 1		0 (0)
Stage 2		212 (45.8)
Stage 3		245 (52.9)
Stage 4		6 (1.3)
Stage 5		0 (0)
**Presence of a stoma**	463	71 (15.3)

ASA: American Society of Anesthesia. ASIA: American Spinal Injury Association. SCI: Spinal cord injury. $^{+}$: As appropriate. $^{++}$: Neurological diseases, such as multiple sclerosis or spina bifida. $^{+++}$: Regardless of SCI.

Table 3-S: Comorbidities.

Comorbidity	n	Total cohort n (%)	Fecal diversion group n = 71	Supported natural defecation group n = 391	*p*-value
Gastrointestinal	462	68 (14.7)	11 (15.5)	57 (14.5)	0.84
Heart	462	69 (14.9)	12 (16.9)	57 (14.5)	0.61
Lung	462	76 (16.5)	12 (16.9)	64 (16.4)	0.91
Hepatic	462	19 (4.1)	5 (7.0)	14 (3.6)	0.18
Renal	462	61 (13.2)	13 (18.3)	48 (12.3)	0.17
Endocrine	462	102 (22.1)	20 (28.2)	82 (21.0)	0.18
Neurological	462	62 (13.4)	13 (18.3)	49 (12.5)	0.19
Psychiatric	462	83 (18.0)	11 (15.5)	72 (18.4)	0.56
Others*	462	336 (72.7)	56 (78.9)	280 (71.6)	0.21

*Urinary tract infection, peripheral artery disease, deep vein thrombosis, hypertension, anemia …

Table 4-S: Medication.

Drug class	n	Total cohort n (%)	Fecal diversion group	Supported natural defecation group	*p*-value
Analgetics (Non-Opioids)	459	112 (24.4)	21 (29.6)	91 (23.5)	0.27
Opioids	460	64 (13.9)	6 (8.5)	58 (14.9)	0.15
Laxatives (osmotic)	460	111 (24.1)	18 (25.4)	93 (23.9)	0.79
Other Laxatives	460	75 (16.3)	8 (11.3)	67 (17.2)	0.21
Klysma	460	50 (10.9)	3 (4.2)	47 (12.1)	0.05
Baclofen	460	173 (37.6)	24 (33.8)	149 (38.3)	0.47
Prokinetics	459	25 (5.4)	3 (4.2)	22 (5.7)	0.62
Probiotics	459	107 (23.3)	14 (19.7)	93 (24.0)	0.55
Anticholinergics	461	217 (47.1)	33 (46.5)	184 (47.2)	0.91

*: Significant with $p < 0.05$. **: Significant with $p < 0.01$.

Table 5-S: Laboratory parameters.

		Number (%) or Median (IQR)+			
		n	Fecal diversion group	Supported natural defecation group	*p*-value++
Before surgery	RBC	458 (68/390)	10.9 (9.1; 12.2)**	12.1 (10.5; 13.6)	0.00005
	WBC	458 (68/390)	8.8 (7.1; 11.6)	9.8 (7.6; 13.1)	0.12
	Platelets	457 (68/389)	339.5 (267.8; 496.3)	330 (245; 428)	0.12
	CRP	458 (68/390)	7.8 (3.1; 14)	7.9 (2.4; 17)	0.97
At discharge	RBC	444 (69/375)	11.2 (10.4; 13.1)**	12.2 (10.9; 13.6)	0.007
	WBC	443 (69/374)	7.1 (5.8; 8.9)	7.1 (5.6; 8.7)	0.67
	Platelets	443 (69/374)	261 (191.5; 331)	252.5 (198; 308.5)	0.92
	CRP	444 (69/375)	1.7 (0.6; 4.4)*	1.2 (0.4; 2.8)	0.02

RBC: Red blood cell count, WBC: White blood cell count, CRP: C-reactive protein. +: As appropriate. ++: Chi2 Test or Mann–Whitney U test, as appropriate. *: Significant with $p < 0.05$. **: Significant with $p < 0.01$.

Table 6-S: Stoma characteristics.

	n	Number (%) or Median (IQR)+
**Presence of a stoma**	71	71 (100)
End colostomy		26 (36.6)
Loop ileostomy		17 (23.9)
Unknown		28 (39.4)
**Time between stoma surgery and current ulcer** [months]	29	40 (2.3; 156.1)
**Adverse event during stoma surgery***	44	11 (25)

+: As appropriate. *Kind of adverse events: Perforation of the colon, wound infection, suture insufficiency, tear out the stoma, wound healing disorder in the area of the abdominal wall, recurrent stoma complications, acute abdomen, sepsis, sigma elongatum, ileus.

Table 7-S: Ulcer characteristics.

Parameter	n	Number (%) or Median (IQR)+
**Size** [cm²]	332	16.0 (8.0; 30.0)
**Severity**	463	
Stage 2		0 (0)
Stage 3		291 (62.9)
Stage 4		172 (37.1)
**Previous surgery**	463	246 (53.1)
Number of surgical procedures ≤ 3		201 (81.7)
Number of surgical procedures > 3		45 (18.3)
**Admission to surgery time** [days]	240	60 (27.3; 140)
**Primary microbial infection**	463	434 (93.7)
**Microbial superinfection**	463	95 (20.5)
**Time until complete cure** [days]	445	61 (44; 89)
**Length of hospital stay** [days]	461	60 (44; 89.5)
**Length of ICU stay** [days]	463	4 (2.75; 22)
**Adverse events** [Ulcer]	463	245 (53.9)
**Adverse events in total**	463	269 (58.1)
**Recurrence** [within 3 months]	463	30 (6.5)
**New ulcer in the same region** [within 3 months]	463	13 (2.8)

ICU: Intensive care unit. +: As appropriate.

Figure 1-S: Comorbidities of the fecal diversion and supported natural defecation group were not significant.

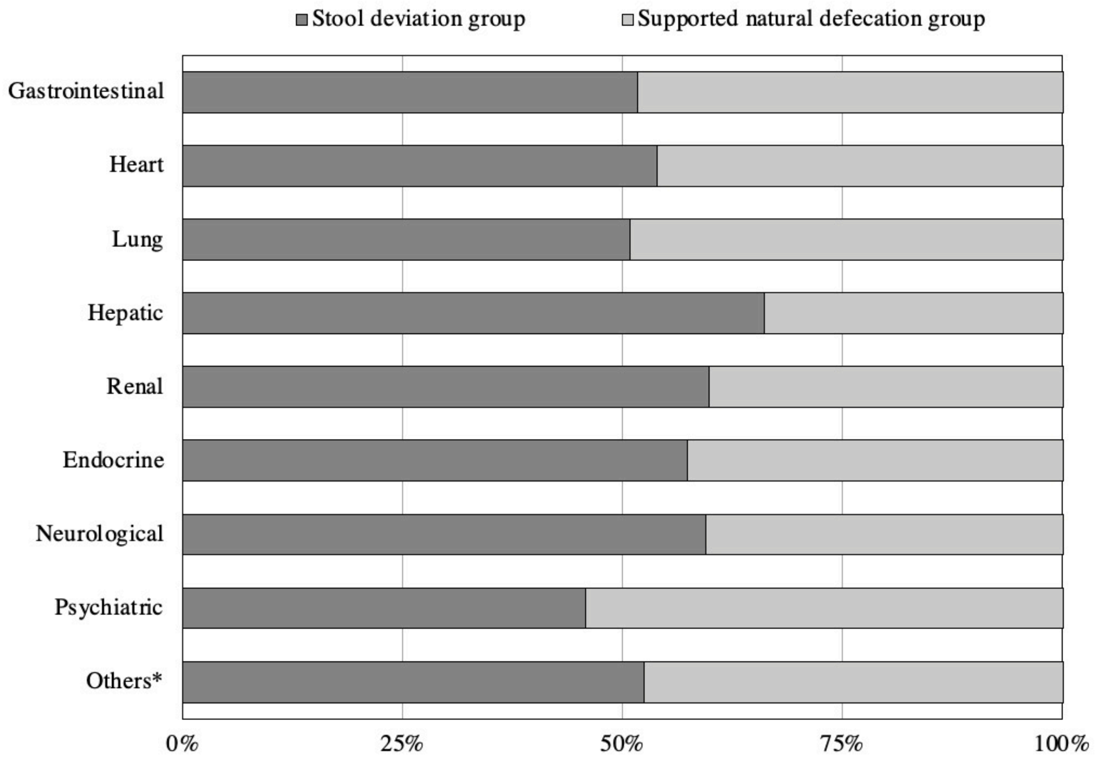

*Urinary tract infection, peripheral artery disease, deep vein thrombosis, hypertension, anemia, etc.

Figure 2-S: ASA stage 3 and 4 were highly significant in patients with fecal diversion.

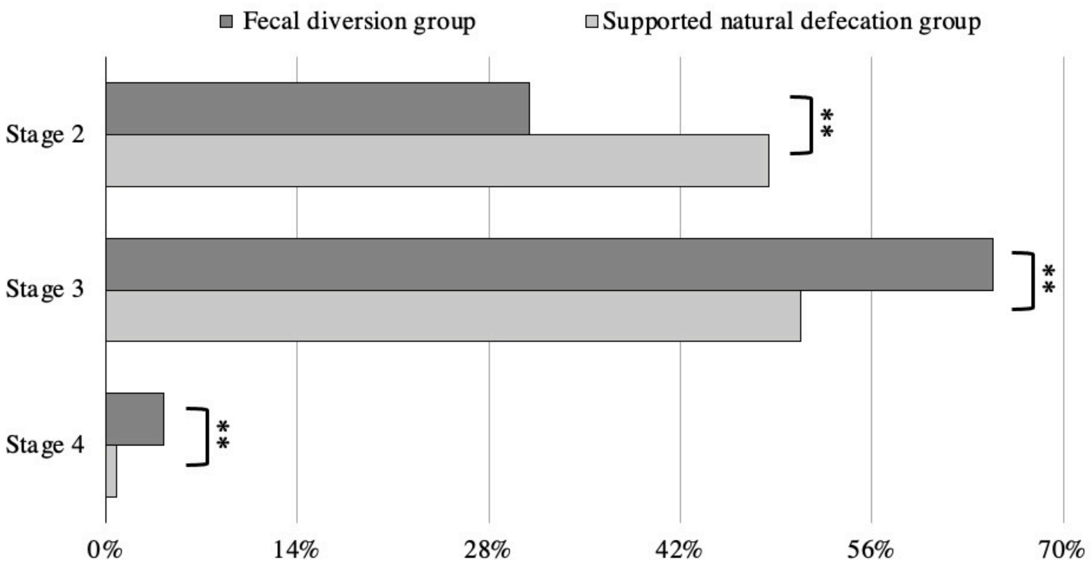

(**p < 0.01). Stage 3 was significant in the natural defecation group (*p < 0.05).
